# Sensitive, High-Throughput Liquid Chromatography-Tandem Mass Spectrometry Analysis of Atorvastatin and Its Pharmacologically Active Metabolites in Serum for Supporting Precision Pharmacotherapy

**DOI:** 10.3390/molecules26051324

**Published:** 2021-03-02

**Authors:** Gellért Balázs Karvaly, István Vincze, István Karádi, Barna Vásárhelyi, András Zsáry

**Affiliations:** 1 Department of Laboratory Medicine, Semmelweis University, Nagyvárad tér 4, H-1089 Budapest, Hungary; vincze.istvan@pharma.semmelweis-univ.hu (I.V.); vasarhelyi.barna@med.semmelweis-univ.hu (B.V.); 2 Department of Internal Medicine and Hematology, Semmelweis University, Szentkirályi út 46, H-1088 Budapest, Hungary; karadi.istvan@med.semmelweis-univ.hu (I.K.); zsary.andras@med.semmelweis-univ.hu (A.Z.)

**Keywords:** atorvastatin, hydroxyatorvastatin, atorvastatin lactone, therapeutic drug monitoring, pharmacokinetics, individualized drug therapy, precision pharmacotherapy

## Abstract

The antihyerlipidemic drug atorvastatin (ATR) is used worldwide as part of the strategy to prevent cardiovascular events. The high prevalence of patient nonadherence remains an important challenge which could be addressed efficiently by precision pharmacotherapy based on therapeutic drug monitoring (TDM). ATR is metabolized to pharmacologically active metabolites, and evidence shows that the sums of ATR acid and lactone form concentrations (ATR + ATRL), or of ATR and hydroxylated metabolites (ATR + MET) should be assayed. A method is presented for the analysis of these substances in serum. Method validation included the estimation of the quantitative relationship between the concentrations and the standard deviations (SD), which supports the optimal incorporation of TDM results into nonparametric pharmacokinetic models. The concentrations of the analytes were evaluated in human subjects receiving ATR. The method’s performance improved by taking the sums of acid and lactone concentrations into account. The concentration–SD relationship was linear, and we recommend applying Theil’s regression for estimating the assay error. All analytes could be detected by 2 h post dose in the samples of human subjects. The changes in metabolite/parent drug concentration ratios in time depended on the dose. The method is suitable for the TDM of ATR with a focus on precision pharmacotherapy.

## 1. Introduction

Atorvastatin (ATR) is an antilipidemic medication which is among the most prescribed drugs worldwide. It exerts its clinical effects by the competitive inhibition of the 3-hydroxy-3-methylgutaryl coenzyme A reductase (Enzyme Commission number 1.1.1.88) and, consequently, by blocking de novo cholesterol synthesis. The main challenge regarding ATR therapy is the high prevalence of patient nonadherence, measures against which are warranted [1,2,3,4]. In addition, acute and chronic adverse conditions, notably, statin-associated muscle symptoms (SAMS), statin-induced autoimmune necrotizing myopathia and statin-induced diabetes present risks associated with ATR therapy. SAMS conditions are the most frequent causes of the withdrawal of statins, which lead to a clinically disadvantageous rise of cardiovascular risk. While currently not part of routine clinical care, therapeutic drug monitoring (TDM) of systemic exposure to ATR can be very useful in revealing if the drug is taken as prescribed.

**Figure 1:**
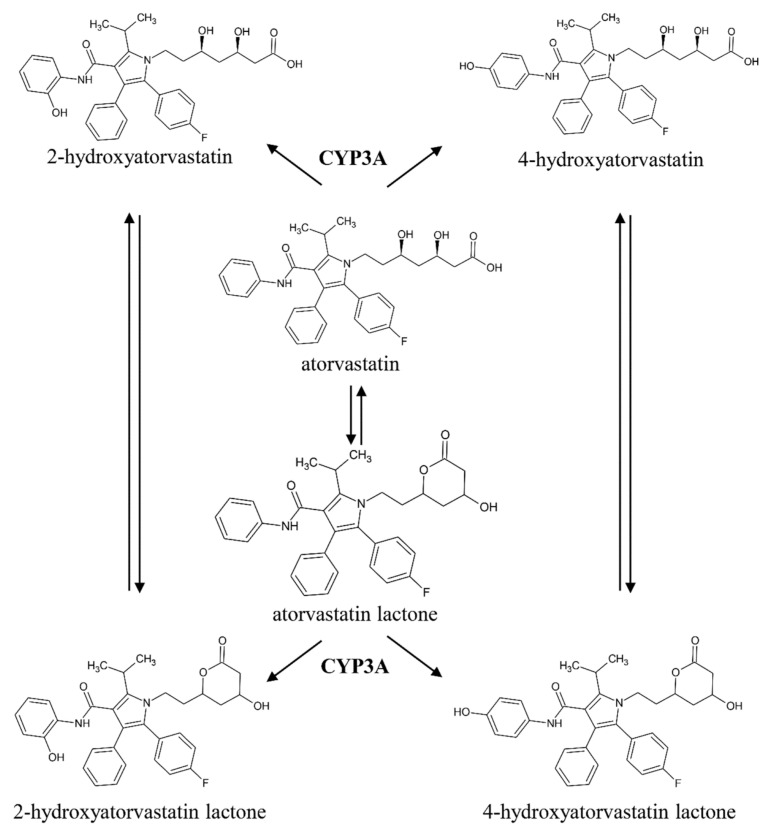
Overview of atorvastatin metabolism. The production of hydroxylated metabolites is catalyzed by cytochrome P450 3A isoenzymes. (CYP3A). The acid and the lactone forms of the parent drug and the metabolites are in equilibrium in serum.

ATR undergoes extensive metabolism including the formation of 2-hydroxy-atorvastatin (2OATR) and 4-hydroxyatorvastatin (4OATR), which are responsible for the majority of ATR-related clinical effects [5]. After its intake, ATR is present both in its acid and lactonized forms in the gastrointestinal tract as well as in the bloodstream as a result of a rapid interconversion [6]. This conversion also applies to its pharmacologically active metabolites (Figure 1) [7]. Consequently, it has been proposed that exposure to ATR is best characterized by the sums of the concentrations of the acid and lactone forms of the parent drug (ATR + ATRL), or of ATR, 2OATR and 4OATR (ATR + MET) [8,9].

Nonparametric pharmacokinetic modeling (NPK) based on the application of the nonparametric adaptive grid (NPAG) algorithm has been a popular approach to construct population models and to target individually established future serum drug concentrations by making adjustments to the dosage regimen. The impact of NPK on precision pharmacotherapy has been demonstrated with a wide range of drugs including immunosuppressants, anti-infective drugs, busulfan, valproate and digoxin [10,11]. However, multiple sources of error, including the inaccuracy of the recorded time of dose intake and of sample collection and errors associated with the assay itself should be taken into account in order to perform weighted nonlinear regression underlying the pharmacokinetic models by using optimal weights, i.e., the reciprocal of the variance. Since the sources of error which occur before the samples arrive at the TDM laboratory cannot be controlled, the reciprocal of the variance which can be coined to each data point in the model is estimated by evaluating the SD of the assayed concentrations, and by applying an additive (termed as lambda) or a multiplicative (termed as gamma) constant for representing the uncontrollable sources of error in the framework of Pmetrics^TM^. Consequently, assay imprecision corresponding to each measured concentration is a crucial piece of information [12]. It has been shown that most bioanalytical methods are heteroscedastic, therefore, the assay error can be approximated by modeling the quantitative relationship between the measured drug concentration and the SD associated with it, e.g., as part of method validation. This eliminates the need for running several repeats of each patient sample which would be highly impractical and cost intensive [12,13]. It has been demonstrated that the concentration–SD relationship is linear for most bioanalytical methods based on the use of liquid chromatography-tandem mass spectrometry (LC-MS/MS), and a methodology has been elaborated for developing the respective quantitative approximation, i.e., the assay error equation [14,15].

Recent advancement in the TDM methodology of ATR indicates that ATR + ATRL and ATR + MET concentrations could be clinically more related to ATR therapy than the levels of individual ATR-related substances [8,9]. We present a sensitive, high-throughput LC-MS/MS method for the simultaneous assessment of ATR, ATRL, 2OATR, 2OATRL, 4OATR and 4OATRL concentrations, compare the goodness of assay error equations established using parametric weighted and unweighted, as well as nonparametric regression for future incorporation into NPK models of ATR, and demonstrate the utility of the developed methodology on serum samples obtained from human subjects having received a single dose of ATR.

## 2. Results

**Figure 2:**
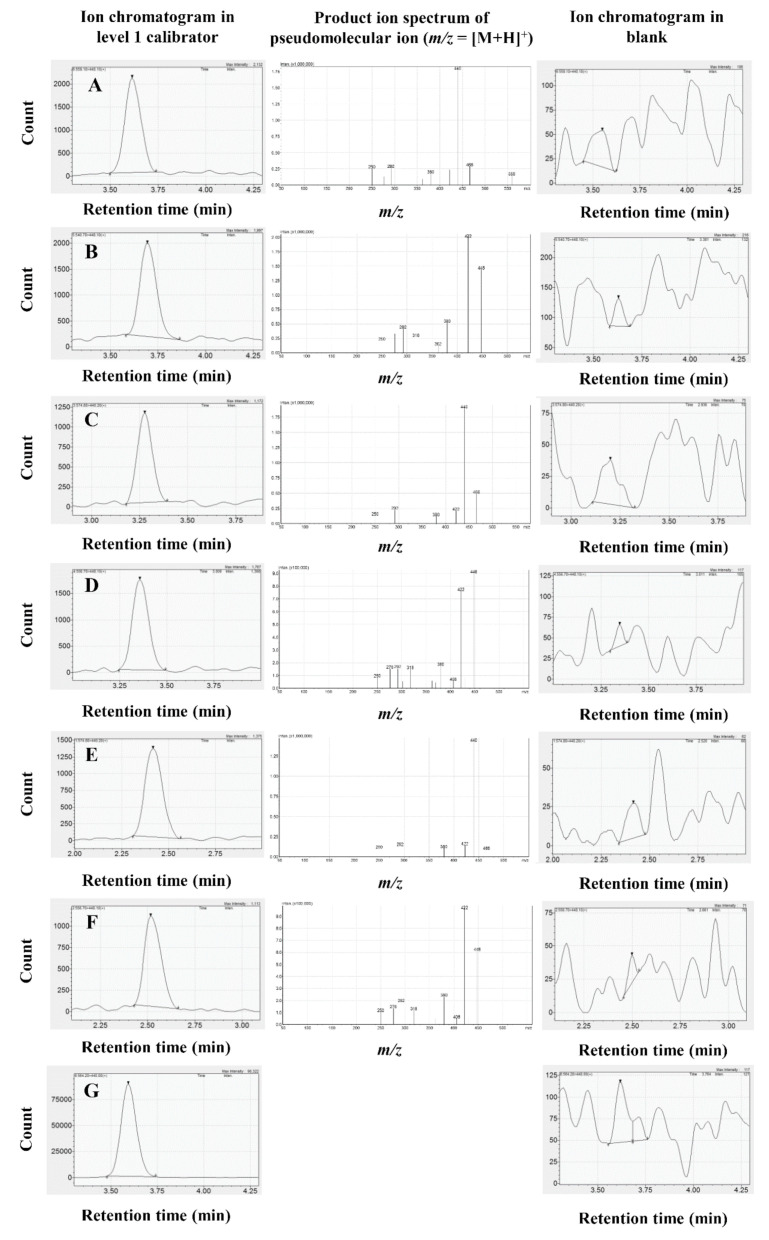
Representative ion chromatograms and product ion mass spectra of the analytes. The product ion mass spectra were recorded between *m/z* = 50 and *m/z* = [M + H]^+^, and the collision energy was −25 V. (**A**) Atorvastatin (1.79 nmol/L), (**B**) Atorvastatin lactone (1.85 nmol/L), (**C**) 2-hydroxyatorvastatin (1.87 nmol/L), (**D**) 2-hydroxyatorvastatin lactone (1.80 nmol/L), (**E**) 4-hydroxyatorvastatin (1.74 nmol/L), (**F**) 4-hydroxyatorvastatin lactone (1.80 nmol/L), (**G**) ^2^H_5_-atorvastatin (internal standard).

Representative ion chromatograms of the 6 analytes and the internal standard (IS), obtained by running the lowest calibrator sample and blank serum, are shown in Figure 2. In the product ion spectra, the base peak of the acid forms (ATR, 2OATR and 4OATR) was *m*/*z* = 440, which lent itself to be chosen as the target product ion. Two intensive ions (*m*/*z* = 448 and *m*/*z* = 422) were obtained in the case of the lactone forms (ATRL, 2OATRL and 4OATRL), with the former selected arbitrarily as the product ion (Figure 2). All analytes and the IS were detected with good selectivity, and no carry-over was observed between samples. ATR-, 2OATR- and 4OATR-related entities were separated well with retention times of 2.4 min (4OATR), 2.5 min (4OATRL), 3.2 min (2AOTR and 2OATRL), 3.6 min (ATR) and 3.7 min (ATRL), respectively. The acid and lactone forms were not resolved chromatographically, but the mass differences of 18 units allowed their completely selective analysis. The limit of detection (LOD) was 0.05 fmol for each analyte. The 6 point calibration models were linear. None of the calibration points had to be excluded due to a back-calculated accuracy outside the 85–115% (80–120% at the LLOQ) range. The intercepts were not tested for the significance of their difference from 0. Detailed information on 3 calibration models obtained on 3 separate days of analysis, evaluated as proposed by the respective bioanalytical method validation guideline of the European Medicines Agency, are displayed in Table 1 [16].

**Figure 3:**
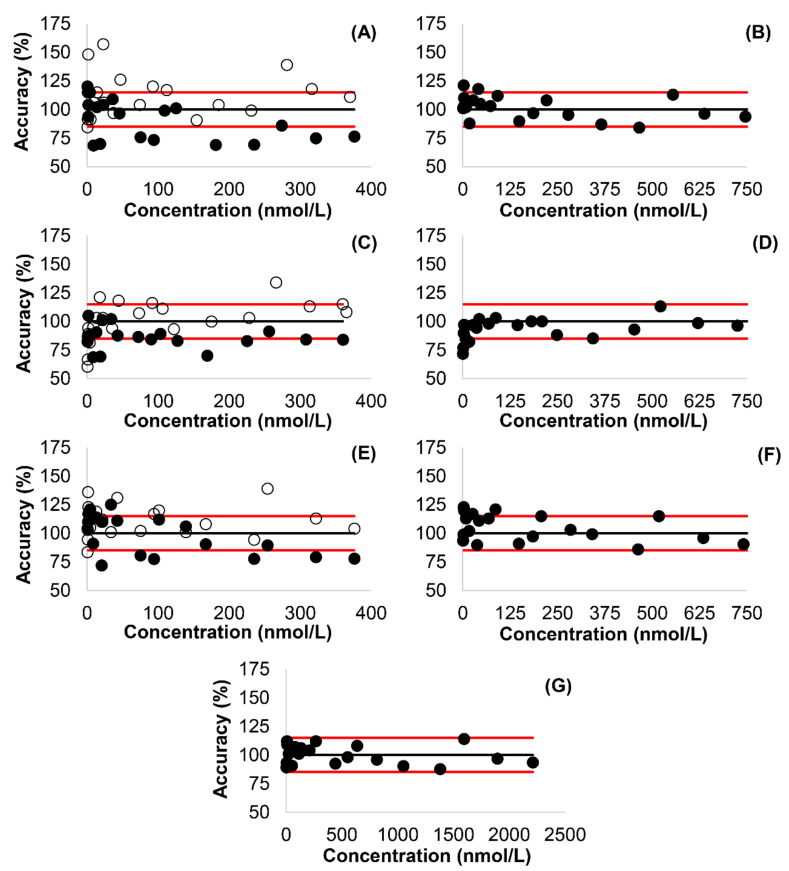
Accuracy of the presented method regarding individual analytes, sums of the concentrations of acid and lactone forms (ATR + ATRL), and the sums of the concentrations of all analytes (ATR + MET). Results obtained at 20 spiking levels, and by spiking 20 independent serum samples at each spiking level, are shown. The horizontal lines indicate an accuracy of 100% (black), 85% and 115% (red). (**A**) Atorvastatin (filled circles) and atorvastatin lactone (hollow circles). (**B**) Combined concentrations of atorvastatin and atorvastatin lactone (ATR + ATRL). (**C**) 2-hydroxyatorvastatin (filled circles) and 2-hydroxyatorvastatin lactone (hollow circles). (**D**) Combined concentrations of 2-hydroxyatorvastatin and 2-hydroxyatorvastatin lactone (2OATR + 2OATRL). (**E**) 4-hydroxyatorvastatin (4OATR) (filled circles) and 4-hydroxyatorvastatin lactone (4OATRL) (hollow circles). (**F**) Combined concentrations of 4-hydroxyatorvastatin and 4-hydroxyatorvastatin lactone (4OATR + 4OATRL). (**G**) Combined concentrations of all analytes (ATR + MET).

Figure 3 shows the trends of accuracy in response to increasing concentrations. The accuracy of ATR, ATRL, 2OATR, 2OATRL, 4OATR, 4OATRL, ATR + ATRL, 2OATR + 2OATRL, 4OATR + 4OATRL, and ATR + MET results was 68.6–120%, 84.4–157%, 68.6–105%, 60.4–134%, 71.9–125%, 83.4–139%, 84.1–121%, 71.6–113%, 86.0–123%, and 87.6–114%, respectively. The accuracy obtained for individual analytes was within the 85–115% range accepted by leading international bioanalytical method validation guidelines at 11–13 of the 20 spiking levels, depending on the analyte [16]. When the combined concentrations of the acid and lactone forms were considered, accuracy was within the limits at 16 (2OATR + 2OATRL, 4OATR + 4OATRL) and at 17 (ATR + ATRL) concentration levels. Finally, the accuracy of ATR + MET results was within the limits of acceptance in all cases. Precision of the assay regarding individual analytes, the combined acid and lactone forms, and ATR + MET was 2.9–21.6%, 2.6–13.8%, and 2.5–7.0%, respectively. The detailed accuracy and the precision values are presented in Tables S1 and S2.

The mean IS-corrected matrix factors were 95.7–111% and 96.6–110% at the low (13.9–14.8 nmol/L) and at the high (13–148 nmol/L) spiking levels, respectively, indicating that the application of ^2^H_5_-atorvastatin as the IS allowed for the correction of matrix effects (Table S3).

**Figure 4:**
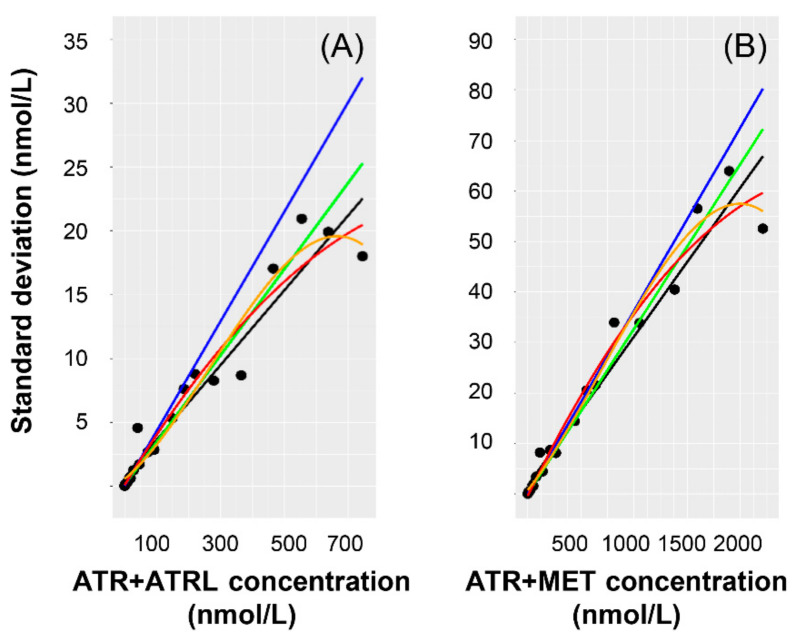
Scatter plots of the standard deviations (SD) of measurement results as plotted against the combined nominal concentrations of (**A**) ATR + ATRL and (**B**) ATR + MET. ATR, ATRL, 2OATR, 2OATRL, 4OATR and 4OATRL were spiked to 20 independent blank serum samples at 20 spiking levels (detailed in Supplementary Table S2). Each data point shows the SD of the combined concentrations. The fitted lines and curves have been obtained by applying various linear and nonlinear regression algorithms to the data points. Black, unweighted linear least squares regression. Blue, weighted linear least squares regression with 1/concentration^2^ weights. Green, Theil’s regression. Red, 2nd-order unweighted nonlinear least squares regression. Orange, 3rd-order unweighted nonlinear least squares regression.

The performance of various linear and nonlinear regression algorithms applied to estimate the assay error of ATR + ATRL and ATR + MET in a quantitative manner is detailed in Table 2, while the fitted curves are shown in Figure 4. The NSSR values of ATR + ATRL were low and fell in a narrow range (3.6669–7.8302, 3.6669–6.5410 by applying linear regression). The slopes (0.0293–0.0429) and intercepts (0.0000–0.0001) obtained using unweighted and 1/concentration^2^-weighted linear least squares, as well as Theil’s regression without or with the Siegel correction were in good agreement. Concerning ATR + MET, fitting an unweighted 2nd-order polynomial yielded an exceptionally high NSSR (102.3). Various linear regression approaches delivered NSSR’s of 1.5784–5.2886, slopes of 0.0290–0.0363, and intercepts of 0.0000–0.0017.

**Figure 5:**
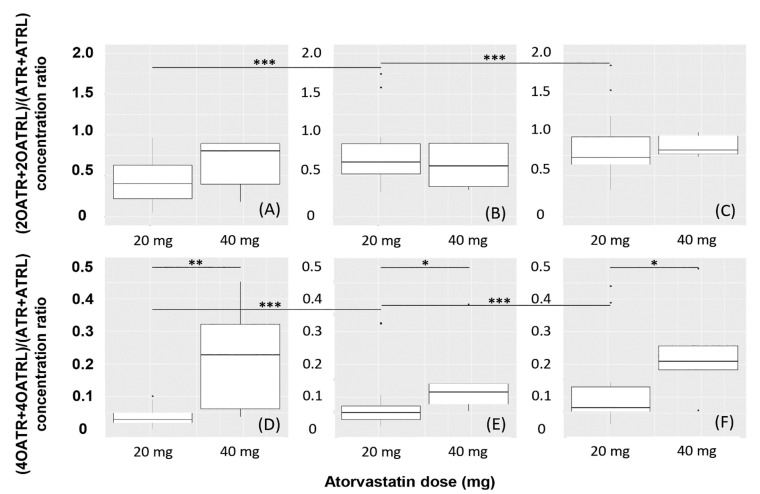
Metabolite/parent drug (acid + lactone form) concentration ratios obtained at 2, 4 and 6 h post dose in human subjects receiving their first 20-mg or 40-mg dose of atorvastatin (n = 29). (2OATR + 2OATRL)/(ATR + ATRL) concentration ratios are displayed at (**A**) 2 h, (**B**) 4 h, and (**C**) 6 h, and (4OATR + 4OATRL)/(ATR + ATRL) concentration ratios at (**D**) 2 h, (**E**) 4 h, and (**F**) 6 h. Asterisks indicate the significance of differences: *p* < 0.05 (*), *p* < 0.01 (**), or *p* < 0.001 (***).

All ATR-related analytes were detected in the blood samples of human subjects receiving their first dose of ATR by 2 h after drug intake (Figure 5). (2OATR + 2OATRL)/(ATR + ATRL) and (4OATR + 4OATRL)/(ATR + ATRL) concentration ratios obtained at 2 h (Figure 5A,D), 4 h (Figure 5B,E), and 6 h (Figure 5C,F) postdose are shown. (4OATR + 4OATRL)/(ATR + ATRL) ratios were statistically higher in subjects taking 40 mg than in those taking 20 mg ATR at all time points (*p* = 0.007, *p* = 0.023 and *p* = 0.030 at 2 h, 4 h and 6 h, respectively, Figure 5D–F). The ratios increased with time (*p* < 0.001 both for 2 h vs. 4 h and for 4 h vs. 6 h concentrations) when the dose was 20 mg, but not when it was 40 mg (*p* = 0.419 and *p* = 0.059, respectively). There was no statistically significant difference between (2OATR + 2OATRL)/(ATR + ATRL) concentration ratios after taking 20 mg or 40 mg atorvastatin at any sampling time (*p* = 0.286, p = 0.544 and *p* = 0.312, respectively, Figure 5A–C). A significant elevation of the ratio over time (*p* < 0.001 and *p* = 0.001 for 2 h vs. 4 h and for 4 h vs. 6 h concentrations, respectively) was observed when 20 mg ATR was taken, but not after administering 40 mg (*p* = 1.000 and *p* = 0.059, respectively).

**Figure 6:**
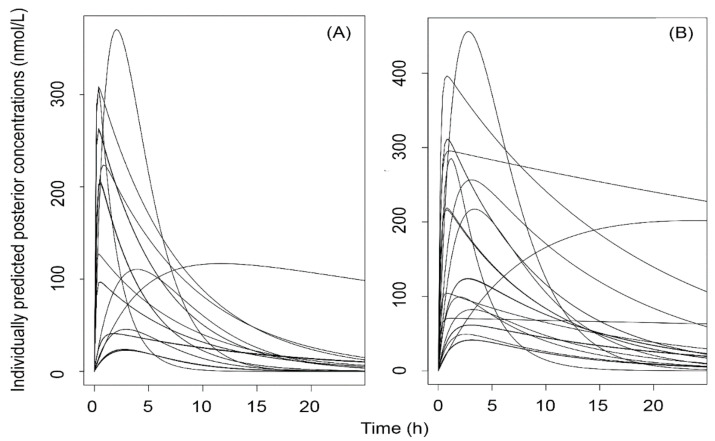
Individual 24 h estimated posterior time-concentration curves obtained by constructing nonparametric population pharmacokinetic models of (**A**) ATR + ATRL and of (**B**) ATR + MET using the software package Pmetrics^TM^.

The measured concentrations of ATR and its metabolites were employed for constructing nonparametric population pharmacokinetic models of ATR + ATRL and of ATR + MET. The individual 24 h posterior ATR + ATRL and ATR + MET pharmacokinetic curves of the 29 subjects included are shown in Figure 6.

## 3. Discussion

There is growing interest in precision pharmacotherapy, and clinical laboratories must adjust their approach to method development and validation in order to be able to support complex planning and evaluation of TDM more efficiently. Several bioanalytical methods have been described for the evaluation of the acid and lactone forms of ATR, 2OATR and 4OATR in serum, and these methods have been reviewed recently [17]. In addition to the validation of the presented method developed for the multiplexed, high-throughput bioanalysis of ATR and its pharmacologically active metabolites, assay error equations have been established to provide important quantitative information on method performance for the construction of nonparametric pharmacokinetic models.

The method proved to be sufficiently selective and sensitive with no interferences or sample carry-over observed. The calibration models showed good linearity across the calibrated concentration range. The accuracy of measurements of individual analytes was, however, not consistently adequate (Figure 3). At low levels, the concentrations of the acid forms of ATR and 4OATR were in most cases slightly overestimated, while those of 2OATR were underestimated. At the same time, all acid forms were consistently underestimated at higher levels. At low levels, lactone forms of ATR and 4OATR delivered similar results with largely overestimated recoveries in some cases, while those of 2OATR were mainly under 100%. Nevertheless, all lactone forms had the tendency to be overestimated in clear contrast to acid forms. When acid and lactone forms were combined, most accuracy values fell in the range of 85–115%, which is commonly accepted by bioanalytical method validation guidelines. This also applied generally to the concentration levels below the lower limit of quantitation. In contrast to individual analytes, no tendency of being over- or underestimated could be observed for the sums of acid and lactone forms. The sums of the concentrations of ATR + MET could be quantitated with acceptable accuracy at all spiking levels.

The RSD’s of individual analyte concentrations spanned a range considerably wider than those of the combined acid and lactone forms, and were, in certain cases, unacceptably large. The RSD’s of the combined acid and lactone forms were acceptable in all cases. The RSD’s of assayed ATR + MET levels were low, remaining conveniently under the proposed cut-off of 15%.

These results indicate the occurrence of an interconversion between the acid and lactone forms in vitro which is difficult to control. Samples were not allowed to stay on the bench for a prolonged time after spiking the analytes, indicating that the interconversion had taken place rapidly. Our findings are the opposite of those described by Vethe et al. who demonstrated that a lactone-to-acid conversion takes place over days [18]. Despite this paradoxical discrepancy, our results afforded very similar conclusions, that is, the combined concentrations of the acid and lactone forms should be evaluated instead of interpreting individual analyte levels considering the fact that the combined levels show adequate stability and their quantitation can be performed with reliable method performance. Interindividual differences in physiological traits and habits (such as nutrition, smoking etc.) could have an important impact on the variability of the interconversion.

The approach to the assessment of method accuracy and precision proposed by bioanalytical method validation guidelines has certain limitations. The obtained values are in fact nothing more than means over nominal concentrations and SD/mean ratios obtained in a very limited number of experiments. Considerably different values may be attained in further independent experiments, and there are no proposals for a follow-up if they do so, with the only aim being to remain within the limits of acceptance. The incorporation of quantitative information on method performance into pharmacokinetic models requires, however, the establishment of assay error at all measured concentrations. Based on statistical theory, the optimal weight for nonlinear least squares regression, the technique employed for estimating pharmacokinetic parameters, is the reciprocal of variance, which can be approximated best by estimating the method-specific SD as a function of the concentration [19]. Regarding the presented approach, the relationship between the concentrations of ATR + ATRL, of ATR + MET levels and of their SD’s was linear. The equations and NSSR’s obtained using various linear regression algorithms were comparable. Since it is the trend of SD’s which needs to be established, Theil’s regression with the Siegel correction is the proposed approach in this respect due to its robustness (Figure 4). Table 2 displays the slopes and the intercepts, i.e., the explicit assay error equations, as well as the NSSR values which serve to describe and compare the sums of the residual errors obtained using the various types of regression.

In the pilot study conducted with the participation of human subjects, the analytes were detected already in the blood samples collected 2 h after the first intake of ATR, indicating that both the absorption and the metabolism of ATR took place rapidly. The observed ranges of (2OATR + 2OATRL)/(ATR + ATRL) concentration ratios showed no relationship with the employed dose at 2, 4 and 6 h. In contrast, differences both in the dose and the time of sampling resulted in differences in (4OATR + 4OATRL)/(ATR + ATRL) concentration ratios. We assume that the production of 2OATR is limited in rate due to the extent of the conversion. Since the formation of 4AOTR is much less intensive, it is also considerably less limited in rate. The dependence of (4OATR + 4AOTRL)/(ATR + ATRL) concentration ratios on the dose could be important in view of reports suggesting a relationship between 4AOTRL levels and SAMS [20,21]. It must be noted that the number of subjects receiving 40 mg ATR was small, posing limitations on our evaluation.

## 4. Materials and Methods

### 4.1. Chemicals and Solutions

Atorvastatin, atorvastatin lactone, 2-hydroxyatorvastatin, 2-hydroxyatorvastatin lactone, 4-hydroxyatorvastatin and 4-hydroxyatorvastatin lactone, as well as the internal standard (IS) ^2^H_5_-atorvastatin were purchased from Alsachim SAS (Illkirch, France). LC-MS grade acetonitrile, formic acid, methanol and water were delivered by Reanal Labor Kft. (Budapest, Hungary).

1 mg/mL stock solutions of the analytes and the internal standard were prepared in methanol. Working solutions were mixes of the analytes at concentrations of 0.04, 0.4, 4 or 40 µmol/L in methanol. The IS working solution contained ^2^H_5_-atorvastatin at 18 µmol/L in methanol. The deproteinizing solution was prepared by adding 70 µL IS working solution to 50 mL acetonitrile.

### 4.2. Biological Samples and Study Design

This work was part of an observational study approved by the Regional and Institutional Committee of Science and Research Ethics, Semmelweis University, Budapest, Hungary (project identifier: 197/2017, date: 2 October 2017). The study was conducted in accordance with the guidelines of the Declaration of Helsinki. All participants gave their informed consent for inclusion prior to beginning their participation. For method development, optimization and validation, as well as for establishing the assay error equations of ATR + ATRL and ATR + MET, residual human serum samples allocated for disposal were obtained from the Central Laboratory (Pest), Department of Laboratory Medicine, Semmelweis University, following their irreversible de-identification. No interaction with patients took place at this stage, no additional blood specimens were collected, and those obtained were not used for any further purpose. Subsequently, samples were obtained from 29 outpatients reporting at the Unit of Cardiology, 3rd Department of Internal Medicine (currently: Department of Internal Medicine and Hematology), Semmelweis University and requiring antihyperlipidemic treatment, but previously not receiving ATR. The subjects took their first dose of 20 mg (n = 24) or 40 mg (n = 5) based on the therapeutic decision made by the clinical recruiting team during their scheduled visit. In a standard phlebotomy process, 2 mL blood was collected from the peripheral vein into native (red-top) phlebotomy tubes immediately before the administration of ATR, and at 2 h, 4 h and 6 h following drug intake. Demographic information of the included human subjects who had given their formal written consent is shown in Table S4.

### 4.3. Sample Preparation

Sample preparation was performed by adding 200 µL Deproteinizing solution to 50 µL serum pipetted onto a Phenomenex Impact 96-well protein precipitation plate (Gen-Lab Kft, Budapest, Hungary), shaking the plate for 10 min at 1100 rpm, forcing the supernatant into a 96-well deep-well collection plate with (2.2 mL/slot) using positive pressure to obtain clean solutions, and diluting 150 µL supernatant with 90 µL LC-MS grade water in another 96-well deep well collection plate.

### 4.4. Analysis

Analysis was performed using liquid chromatography-tandem mass spectrometry. The analytical system consisted of a Shimadzu Nexera X2 modular ultra-high performance liquid chromatographic system coupled to a Shimadzu LCMS-8060 triple quadrupole mass spectrometer (Simkon Kft, Budapest, Hungary). Separation was performed on a Phenomenex Kinetex XB-C18 (50 × 2.1 mm, 1.7 µm) stationary phase (Gen-Lab Kft, Budapest, Hungary) thermostated at 40 °C. The mobile phases were LC-MS grade water and methanol, both of which contained 0.1% formic acid. A 1.0 µL sample was injected. The mass spectrometer was operated using positive electrospray ionization in multiple reaction monitoring mode. The precursor ions, which were the pseudomolecular ions [M + H]^+^ in all cases, as well as the transitions giving rise to the product ions, were selected manually based on the results of full scans and of product ion scans of the 0.5 µg/mL methanolic solutions of the analytes and of the IS, and by considering the high relative intensity of these ions, The optimization of the mass spectrometry settings (quadrupole 1 bias, collision energy and quadrupole 3 bias) was performed automatically by the instrument control software. The retention times and the monitored ion transitions were 3.6 min, 559.1 > 440.1 (target) and 559.1 > 292.1 (qualifier) for ATR, 3.7 min, 540.7 > 448.1 (target) and 540.7 > 422.1 (qualifier) for ARL, 3.3 min, 574.8 > 440.2 (target) and 574.8 > 466.1 (qualifier) for 2OATR, 3.4 min, 556.7 > 448.1 (target) and 556.7 > 422.1 (qualifier) for 2OATRL, 2.4 min, 574.8 > 440.2 (target) and 574.8>250.1 (qualifier) for 4OATR, 2.5 min, 556.7 > 448.1 (target) and 556.7 > 422.1 (qualifier) for 4OATRL, and 3.6 min, 564.2 > 440.0 (target) for the IS. Calibration using samples containing known concentrations of the analytes (1.0, 5.0, 10, 50, 100 and 200 ng/mL, corresponding to 1.74–1.85, 8.70–9.24, 17.4–18.5, 87.0–92.4, 174–185 and 348–370 nmol/L, respectively) was conducted at the beginning of each batch using pooled blank serum, in which the absence of the analytes had been confirmed. The working solutions of the analytes were spiked to the blank serum with volumes not exceeding 5% of that of the serum fraction. Each calibrator and experimental sample was tested in a single repeat.

### 4.5. Method Validation

Selectivity of the method was verified by running solvent blanks (water-methanol 1:1) and the serum specimens subsequently employed for establishing accuracy, precision and the assay error equations, without spiking the analytes to them. Sample carry-over was evaluated by comparing peak intensities obtained at the monitored ion transitions when a blank solution was injected after the highest-level calibrator. The performance of the calibration curves was tested by calculating the determination coefficients and the back-calculated accuracies, and by examining the number of calibrators which had to be omitted from the calibration models on 3 separate days of analysis. No lower limit of quantification (LLOQ) was defined since a central concept of generating assay error equations in support of the nonparametric population pharmacokinetic modeling procedure using the Pmetrics^TM^ package of R is to establish the precision of the method all the way down to zero concentration, as described by Jelliffe et al. [12].

Assay accuracy, assay precision and the assay error equations were established by spiking serum samples (n = 60) previously screened for the absence of the substances of interest with the analytes at 4, 6 and 10 different levels on 3 separate days, resulting in a total of 20 spiking levels (Table S2). Establishing overall assay precision is pivotal when the aim is to support nonparametric pharmacokinetic modeling. This experimental design allowed us to add further variability by applying the developed method to different numbers of serum samples containing the analytes at different concentrations on each day, thereby modeling the assay performance in a more variable application environment instead of testing its reproducibility by using the same sets of samples, which is a more limited approach. Since patient samples are typically assayed once in the clinical laboratory, the results obtained on the 3 days were combined for evaluation instead of conducting separate within-run and between-run experiments, as proposed by [22].

Internal standard-corrected matrix factors (IMF) were calculated at two concentration levels (8.0 and 80 ng/mL corresponding to 13.9–14.8 nmol/L and 139–148 nmol/L depending on the analyte, respectively) by spiking a methanolic solution containing the analytes at 336 ng/mL (0.585–0.622 nmol/L) or 3.36 µg/mL (5.85–6.22 nmol/L), as well as a 2 µmol/L methanolic solution of the IS, 5 µL each, to 140 µL supernatant obtained following deproteinization of 50 µL blank serum with 200 µL acetonitrile as described in the sample preparation section. 10 independent serum matrices were used. 140 µL acetonitrile-water 4:1 (*v*/*v*) mixture was spiked in the same manner, and 150 µL of this mixture was diluted with 90 µL water. The IMF was calculated as shown by Equation (1).

(1) $$IMF\;=\;\frac{peak\;area_{analyte,\;serum\;}\times \;peak\;area_{IS,\;solution}}{peak\;area_{analyte,\;solution}\;\times \;peak\;area_{IS,\;serum}}$$

### 4.6. Data Evaluation

Calculations were made in Shimadzu Labsolutions v5.89, Microsoft Excel and in the R environment [23]. Analyte/IS peak area ratios were calculated for the evaluation of concentrations. Calibration was performed by linear regression using 1/x^2^ weights. Accuracy was the ratio of the mean measured versus the nominal concentration, expressed as a percentage. The SD of the assayed concentrations was determined at each spiking level. Relative standard deviations (RSD) were the ratios of SD and mean measured concentrations, expressed as a percentage. The establishment of the assay error equation has been proposed to include the SD’s of concentrations measured in the blank samples, therefore, these SD’s were also calculated [12,13]. The analyte concentrations measured in the blanks, as well as those found in samples containing the analytes at a level lower than that of the lowest calibrator, were estimated by single-point calibration using the lowest level calibrator.

The ’stats’ package was used for applying various parametric regression algorithms. Unweighted linear, 2nd- and 3rd-order nonlinear least squares have been employed for estimating the assay error of automated clinical analyzers and, in one case, of a high-performance liquid chromatography-ultraviolet absorption detection method [12,13]. Linear least squares regression with 1/concentration^2^ weights has been proposed as the best estimate of the reciprocal of the assay variance, the optimal weight for linear and nonlinear regression, in bioanalytical applications of LC-MS/MS [14]. The ’mblm’ package of R was employed for performing Theil’s regression without (Equations (2) and (3)) or with the Siegel correction (Equations (4) and (5)). Theil’s regression is a nonparametric linear regression approach having the advantage over parametric algorithms that it is unbiased across the fitted concentration range, and is very robust, i.e., it displays the linear trend without being sensitive to the presence of outliers [24,25].

(2) $$slope\;=\;median\;\left[\;\frac{\left(SD_{j}\;-\;SD_{i}\right)}{\left(concentration_{j\;}-\;concentration_{i}\right)}\right]$$

(3) $$intercept\;=\;median\;\left(SD_{i}-slope\cdot concentration_{i}\right)$$

where *i* and *j* denote the *i*-th and *j*-th spiking levels, and *j* > *i*.

(4) $$slope\;=\;median\left[\begin{array}{c} median\\ i\neq j \end{array}\;\frac{\left(SD_{j}\;-\;SD_{i}\right)}{\left(concentration_{j}\;-\;concentration_{i}\right)}\right]$$

(5) $$intercept\;=\;median\left[\begin{array}{c} median\\ i\neq j \end{array}\;\frac{\left(concentration_{j}SD_{i}\;-\;concentration_{i}SD_{j}\right)}{\left(concentration_{j\;}-\;concentration_{i}\right)}\right]$$

where *j* ≠ *i*. To obtain the slope, (*SD_j_* − *SD_i_*)/(*concentrations_j_*–*concentration_i_*) ratios are calculated for each *i*-th and all other spiking levels, and their median is recorded. The overall median of the n recorded medians is subsequently calculated. The intercept is calculated likewise. In Equations (1)–(4) concentrations correspond the nominal concentrations spiked to the serum samples at each *i*-th and *j*-th spiking level.

The goodness of the fit of the regression lines was determined by calculating the sums of squared residuals normalized to the predicted standard deviations (NSSR), an unbiased estimator, as shown in Equation (6).

(6) $$NSSR\;=\;\sum_{i}^{n}\frac{{\left(SD_{obs,i}\;-\;SD_{pred,i}\right)}^{2}}{SD_{pred,i}{}^{2}}$$

where *SD_obs,i_* is the observed *SD* at the *i*-th spiking level, and *SD_pred,i_* is the *SD* estimated by each employed regression algorithm.

## 5. Conclusions

In conclusion, a novel bioanalytical method has been presented for the quantitation of ATR and its pharmacologically active metabolites in human serum. Method performance proved to be acceptable when the combined concentrations of acid and lactone forms were evaluated. The described methodology and the assay error equations established by applying Theil’s regression and the Siegel correction provide an efficient analytical background for nonparametric population pharmacokinetic modeling and for precision pharmacotherapy based on the use of pharmacokinetic models.

## Figures and Tables

**Table 1:** Calibration curve data obtained on 3 separate days. 6 point calibration was employed using weighted linear regression with 1/x^2^-weights. ATR, atorvastatin. ATRL, atorvastatin lactone. 2OATR, 2-hydroxyatorvastatin. 2OATRL, 2-hydroxyatorvastatin lactone. 4OATR, 4-hydroxyatorvastatin. 4OATRL, 4-hydroxyatorvastatin lactone. r^2^, determination coefficient.

Day	Calibration Curve Data		Back-calculated Accuracy (%)	Calibrated Concentration Range (nmol/L)
	Equation	r^2^		
ATR				
1	Y = 0.0157x + 0.0042	0.9990	96.1–104	1.79–358
2	Y = 0.0187x + 0.0047	0.9994	98.1–104	
3	Y = 0.0149x + 0.0028	0.9970	94.4–107	
ATRL				
1	Y = 0.0274x + 0.0009	0.9965	90.4–107	1.85–370
2	Y = 0.0273x + 0.0003	0.9988	94.5–102	
3	Y = 0.0198x + 0.0000	0.9979	93.5–106	
2OATR				
1	Y = 0.0109x + 0.0029	0.9999	98.9–102	1.74–348
2	Y = 0.0085x + 0.0012	0.9983	95.6–105	
3	Y = 0.0115x + 0.0010	0.9997	97.8–102	
2OATRL				
1	Y = 0.0226x + 0.0101	0.9999	98.9–102	1.80–359
2	Y = 0.0169x + 0.0012	0.9983	95.6–105	
3	Y = 0.0153x + 0.0038	0.9982	97.8–102	
4OATR				
1	Y = 0.0185x + 0.0037	0.9973	94.4–106	1.74–348
2	Y = 0.0167x + 0.0006	0.9984	96.1–104	
3	Y = 0.0141x + 0.0007	0.9990	96.4–104	
4OATRL				
1	Y = 0.0191x + 0.0014	0.9952	90.6–110	1.80–359
2	Y = 0.0153x + 0.0011	0.9972	92.4–104	
3	Y = 0.0120x + 0.0013	0.9970	91.2–104	

**Table 2:** Coefficients of the calculated assay error equations and the estimators of the goodness of the fits. ATR, atorvastatin. ATR-L, atorvastatin lactone. 2OATR, 2-hydroxyatorvastatin. 2OATRL, 2-hydroxyatorvastatin lactone. 4OATR, 4-hydroxyatorvastatin. 4OATRL, 4-hydroxyatorvastatin lactone. NSSR, sums of squared residuals normalized to the predicted standard deviations.

	ATR + ATRL	ATR + MET
Unweighted linear least squares		
Intercept	0.0000	0.0017
Linear coefficient (slope)	0.0293	0.0290
NSSR	6.5410	5.2886
Unweighted second-order least squares		
Intercept	0.0001	−0.0004
Linear coefficient	0.0410	0.0434
Second-order coefficient	−0.0184	−0.0073
NSSR	3.7376	102.3
Unweighted third-order least squares		
Intercept	0.0006	0.0007
Linear coefficient	0.0208	0.0271
Second-order coefficient	0.0659	0.0157
Third-order coefficient	−0.0816	−0.0075
NSSR	7.8302	4.4192
1/x^2^-weighted linear regression		
Intercept	0.0000	0.0000
Linear coefficient (slope)	0.0429	0.0363
NSSR	3.6669	1.5784
Theil’s regression		
Intercept	0.0001	0.0000
Linear coefficient (slope)	0.0338	0.0327
NSSR	5.4048	2.1529
Theil’s regression with Siegel correction		
Intercept	0.0001	0.0000
Linear coefficient (slope)	0.0353	0.0332
NSSR	5.4125	2.1363

## Data Availability

Data supporting the presented results can be obtained from the authors upon reasonable request.

## Supplementary Materials

The following are available online at, Table S1: Accuracy and precision obtained for the individual analytes in spiked serum samples, Table S2: Accuracy and precision of the developed method regarding the combined concentrations of the acid and lactone forms of the analytes (ATR + ATRL, 2OATR + 2OATRL, 4OATR + 4OATRL and ATR + MET) in spiked samples, Table S3: Internal standard-corrected matrix factors (IMF) influencing the quantitation of the acid and lactone forms of atorvastatin and its hydroxylated metabolites, Table S4: Demographic information on the subjects included in the study.
