# Supplementary material for: Sensitive, High-Throughput Liquid Chromatography-Tandem Mass Spectrometry Analysis of Atorvastatin and Its Pharmacologically Active Metabolites in Serum for Supporting Precision Pharmacotherapy
Source: Molecules. 2021 Mar 2;26(5):1324. doi: 10.3390/molecules26051324 (PMC7958319; doi:10.3390/molecules26051324)
Supplement: Supplementary file 1 [file molecules-26-01324-s001.pdf]

molecules

MDPI

–Supplementary Material

Sensitive, High-Throughput Liquid Chromatography-Tandem Mass Spectrometry Analysis of Atorvastatin and Its Pharmacologically Active Metabolites in Serum for Supporting Precision Pharmacotherapy

Gellért Balázs Karvaly [1,*], István Vincze [1], István Karádi [2], Barna Vásárhelyi [1] and András Zsáry [2]

[1] Department of Laboratory Medicine, Semmelweis University, Nagyvárad tér 4, H-1089 Budapest, Hungary; karvaly.gellert_balazs@med.semmelweis-univ.hu (G.B.K.); vincze.istvan@pharma.semmelweis-univ.hu (I.V.); vasarhelyi.barna@med.semmelweis-univ.hu (B.V.)
[2] Department of Internal Medicine and Hematology, Semmelweis University, Szentkirályi út 46, H-1088 Budapest, Hungary; karadi.istvan@med.semmelweis-univ.hu (I.K.); zsary.andras@med.semmelweis-univ.hu (A.Z.)
* Correspondence: karvaly.gellert_balazs@med.semmelweis-univ.hu; Tel. +36-1-459-1500/extension 56561

Table S1. Accuracy and precision obtained for the individual analytes in spiked serum samples. 20 spiking levels were used in 3 independent experiments. 20 independent serum samples previously verified not to contain any of the analytes were used in each experiment. ATR, atorvastatin. ATRL, atorvastatin lactone. 2OATR, 2-hydroxyatorvastatin. 2OATRL, 2-hydroxyatorvastatin lactone. 4OATR, 4-hydroxyatorvastatin. 4OATRL, 4-hydroxyatorvastatin lactone. RSD, relative standard deviation.

	ATR			ATRL			2OATR		
Spiking Level	Nominal Concentration (nmol/L)	Accuracy (%)	RSD (%)	Nominal Concentration (nmol/L)	Accuracy (%)	RSD (%)	Nominal Concentration (nmol/L)	Accuracy (%)	RSD (%)
1	0.417	120	12.3	0.514	84.4	15.7	0.423	82.3	15.7
2	0.834	115	9.4	1.03	91.4	15.7	0.846	86.9	10.6
3	1.37	93.7	10.2	1.40	148	7.9	1.28	85.2	12.3
4	1.81	104	9.8	1.85	116	8.8	1.69	105	7.0
5	3.76	115	10.2	4.62	91.4	9.0	3.81	88.0	6.6
6	9.00	68.6	9.8	9.25	106	6.1	8.46	68.6	7.5
7	13.7	102	7.9	14.0	115	6.8	12.8	90.3	5.9
8	18.3	69.8	13.4	22.6	157	12.5	18.6	69.2	9.2
9	22.7	104	8.4	23.3	106	6.5	21.4	101	6.0
10	36.0	109	7.0	37.0	96.9	13.1	33.8	102	4.8
11	45.5	96.5	4.5	46.8	126	5.7	42.8	87.6	4.5
12	75.2	75.6	3.8	74.0	104	5.9	71.9	86.4	2.9
13	93.8	73.3	4.9	92.5	120	5.1	90.0	84.2	4.0
14	109	99.0	6.1	112	117	8.0	103	89.1	4.8
15	125	101	6.9	154	90.6	10.6	127	82.9	4.1
16	181	69.0	5.2	185	104	7.0	169	69.9	3.5
17	235	69.2	3.2	231	99.1	8.2	225	82.8	3.1
18	274	85.9	5.0	281	139	4.7	256	91.1	4.5
19	322	74.9	4.5	316	118	4.5	308	84.1	3.8
20	376	76.3	3.9	370	111	4.3	360	83.8	3.9
	2OATRL			4OATR			4OATRL		
1	0.406	60.4	17.4	0.465	104	13.3	0.485	83.4	18.1
2	0.810	66.7	10.4	0.929	103	13.4	0.972	94.6	21.6

3	1.33	94.2	13.5	1.27	110	7.2	1.32	136	7.1
4	1.75	89.1	12.4	1.67	117	13.9	1.75	123	13.4
5	3.65	81.4	8.9	4.18	121	9.3	4.37	105	15.7
6	8.75	95.1	5.8	8.35	91.0	9.7	8.75	113	11.2
7	13.3	103	8.2	12.7	114	6.7	13.3	119	6.2
8	17.8	121	9.4	20.4	71.9	17.4	17.8	110	17.0
9	22.1	103	6.6	21.1	110	6.1	22.1	112	4.9
10	35.0	93.8	12.2	33.4	125	9.2	35.0	101	15.7
11	44.2	118	5.3	42.3	111	4.4	44.2	131	6.1
12	72.9	107	4.6	75.2	80.6	3.2	72.9	102	7.0
13	91.3	116	5.4	94.0	77.5	4.3	91.1	117	5.1
14	106	111	7.5	101	112	5.3	106	120	6.7
15	122	93.2	8.8	139	106	8.2	146	101	17.0
16	175	99.8	7.3	167	90.5	10.0	175	108	13.6
17	228	103	7.3	235	77.7	4.1	228	94.5	5.7
18	266	134	4.9	254	89.4	4.6	264	139	4.3
19	313	113	4.4	322	79.2	5.2	313	113	5.8
20	365	108	4.6	376	77.8	4.6	365	104	4.1

Table S2. Accuracy and precision calculated for the combined concentrations of the acid and lactone forms of the analytes (ATR + ATRL, 2OATR + 2OATRL, 4OATR + 4OATRL and ATR + MET) in spiked samples. 3 separate experiments were performed. ATR, atorvastatin. ATRL, atorvastatin lactone. 2OATR, 2-hydroxyatorvastatin. 2OATRL, 2-hydroxyatorvastatin lactone. 4OATR, 4-hydroxyatorvastatin. 4OATRL, 4-hydroxyatorvastatin lactone. RSD, relative standard deviation.

Spiking Level	ATR+ATRL			2OATR+2OATRL			4OATR+4OATRL			ATR+MET		
	Nominal Concentration (nmol/L)	Accuracy (%)	RSD (%)	Nominal Concentration (nmol/L)	Accuracy (%)	RSD (%)	Nominal Concentration (nmol/L)	Accuracy (%)	RSD (%)	Nominal Concentration (nmol/L)	Accuracy (%)	RSD (%)
1	0.931	101	9.0	0.829	71.6	9.6	0.950	93.6	7.0	2.71	89.2	4.4
2	1.86	102	8.7	1.66	77.0	7.8	1.90	98.9	8.8	5.42	93.2	7.0
3	2.77	121	5.4	2.61	89.7	7.9	2.59	123	5.0	7.97	112	3.3
4	3.66	110	4.7	3.45	97.0	5.0	3.42	120	4.1	10.5	109	3.5
5	8.38	102	3.8	7.46	84.8	4.6	8.54	113	3.8	24.4	101	3.0
6	18.3	87.7	4.0	17.2	82.1	4.5	17.1	102	5.0	52.6	90.6	3.3
7	27.7	108	4.2	26.1	96.7	5.4	25.9	117	4.4	79.7	107	4.1
8	40.8	118	9.5	36.4	94.4	8.1	38.1	89.7	13.8	115	101	7.0
9	46.0	105	3.6	43.5	102	3.9	43.2	111	3.4	133	106	3.2
10	73.0	103	3.6	68.8	97.9	5.0	68.4	113	4.0	210	104	3.9
11	92.3	112	2.8	87.0	103	2.9	86.5	121	3.8	266	112	2.7
12	149	89.8	4.0	145	96.7	2.9	148	91.0	4.7	442	92.4	3.5
13	186	96.7	4.2	181	100	4.2	185	97.2	4.0	553	98.1	3.8
14	221	108	3.7	209	100	4.1	207	115	3.3	637	108	3.1
15	279	95.3	3.1	249	88.0	4.1	285	103	6.3	813	95.9	4.4
16	366	86.9	2.7	345	85.1	3.8	342	99.3	5.6	1052	90.3	3.6
17	466	84.1	4.4	453	92.8	3.8	463	86.0	3.4	1382	87.6	3.4
18	555	113	3.4	522	113	3.8	518	115	3.6	1595	114	3.1
19	639	96.2	3.2	621	98.5	3.2	635	95.9	5.1	1894	96.8	3.5
20	746	93.7	2.6	725	96.2	3.0	741	90.5	3.4	2211	93.5	2.5

Table S3. Internal standard-corrected matrix factors (IMF) influencing the quantitation of the acid and lactone forms of atorvastatin and its hydroxylated metabolites.

Analyte	Level 1		Level 2	
	**Concentration (nmol/L)**	**IMF (%)**	**Concentration (nmol/L)**	**IMF (%)**
atorvastatin	14.3	107 ± 4.3	143	110 ± 4.7
atorvastatin lactone	14.8	95.7 ± 4.7	148	96.6 ± 5.7
2-hydroxyatorvastatin	13.9	97.1 ± 6.1	139	102 ± 4.6
2-hydroxyatorvastatin lactone	14.4	102 ± 4.4	144	101 ± 5.7
4-hydroxyatorvastatin	13.9	111 ± 4.6	139	104 ± 4.6
4-hydroxyatorvastatin lactone	14.4	98.7 ± 4.3	144	97.2 ± 5.4

Table S4. Demographic information on the subjects included in the study.

**(A)**				
		**Total**	**Female**	**Male**
Number of subjects (n)		29	9	20
Age (years)		76 (29 − 88)	76 (29–84)	76 (45–88)
BMI		28.1 (17.1 − 43.4)	28.8 (17.1–37.3)	27.0 (18.6–43.4)
LDL cholesterol (mmol/L)		2.31 (1.24 − 4.50)	3.68 (1.25–4.15)	2.23 (1.24–4.50)
HDL cholesterol (mmol/L)		0.92 (0.58 − 1.72)	0.95 (0.77–1.53)	0.91 (0.58–1.72)
Triglycerides (mmol/L)		1.12 (0.43–24.3)	1.27 (0.85–4.08)	1.07 (0.43–24.3)
Total cholesterol (mmol/L)		3.5 (1.3–12.2)	5.4 (1.3–6.5)	3.4 (2.2–12.2)
Creatinine kinase (U/L)		96 (15–224)	126 (65–215)	94 (15–224)
GPT (U/L)		22 (12–221)	29 (17–37)	27 (19–43)
GOT (U/L)		26 (12–101)	26 (15–101)	25 (12–52)
LDH (U/L)		192 (98–356)	250 (200–356)	168.5 (98–313)
Se. creatinine (μmol/L)		88 (47–212)	70 (47–140)	102 (58–212)
Atorvastatin dose (n)	20 mg	24	7	17
	40 mg	5	2	3
